# Where did civil servants go? the effect of an increase in public co-payments on double insured patients

**DOI:** 10.1186/s13561-016-0093-7

**Published:** 2016-05-12

**Authors:** Sofia Vaz, Pedro Ramos

**Affiliations:** 1 Faculdade de Economia, Universidade do Porto, Porto, Portugal; 2 Administração Regional de Saúde do Norte, Porto, Portugal; 3 Hospital das Clínicas da Faculdade de Medicina da Universidade de São Paulo, Universidade de São Paulo, Av. Dr. Enéas de Carvalho Aguiar, 255, Cerqueira César, São Paulo 05403-000 Brazil

**Keywords:** Health subsystem, Co-payments, National health service, Double coverage

## Abstract

In Portugal, Civil Servants may have a differential utilization of health services due to their supplementary Health Subsystem (ADSE), which grants them access to health services in the private sector at lower price. We exploit the impact of this double coverage on the demand for Portuguese Public Emergency Departments (ED), following the recent increase in co-payments for public health care services in Portugal.

Using detailed ED level data from three different EDs, one for each level of the Portuguese ED care, we rely on a difference-in-differences strategy, under the assumption that both civil servants and National Health Service (NHS) users were targeted by the public co-payment increase, but just the former have a low-cost alternative in the private sector that they can use when prices increase in the NHS.

We found that the existence of a low-price alternative in the private sector caused ED demand to decrease among ADSE beneficiaries following a policy that increased co-payments in public NHS hospitals. Specifically, we show that this decrease was only significant for conditions which have arguably the closest substitutes in the private sector – the low and intermediate-severity conditions – and to patients who lived closer to the ED and to whom the co-payment was the largest share of the ED visit cost.

These findings cast some concerns over the equity of the Portuguese Health System, since civil servants increasingly opt out from public health services but must co-fund both the ADSE and the NHS.

## Background

In several countries with universal National Health Service (NHS) funded by taxation, some public or private subsystems, mutual funds or insurance schemes financed by a mix of employers and employees’ contributions subsist. In the vast majority of these countries, opting out from the NHS is not possible and, therefore, these health subsystems represent a secondary layer of coverage, on top of the NHS, which usually grants access to a variety of services in the private sector [1, 2].

There is nowadays a vast plethora of research that underlines a positive association between additional insurance coverage and healthcare utilization [3–8]. In Portugal, some researchers [9, 10] have found that the double coverage that beneficiaries of the Civil Servants’ Health Subsystem (ADSE) are entitled to increases their probability of using health services, particularly in the private sector where their insurance has a network of preferential providers at a lower cost.

To our knowledge, however, any differential behavior on the demand for care among these double insured patients when co-payments for the public sector increase has never been reported. Economic theory predicts that these patients may be more cost-sensitive to co-payments’ increases in the NHS because they have a low-cost alternative in the private sector that they can use when prices increase in the NHS. Besley and Coate [11] were the firsts to argue that the public provision of healthcare may be redistributive when low income patients resort to public facilities, whereas high income citizens, who contribute to subsidizing the public services through income taxes, opt out to the private sector. Recently, this hypothesis that double insured individuals free up public resources by opting out from the NHS has been tested by several authors: in the Italian voluntary health insurance market, Fabbri and Monfardini [12] found that double insured patients consumed more private services while simultaneously reduced public demand, as did Gertler and Sturm in Jamaica [13]; and in Denmark, Sogaard and colleagues reported that employer-paid health insurance led to a 10 % reduction of public hospitals and substitution with private hospitals [14].

We take advantage of a recent policy that increased co-payments in the Portuguese NHS to test this hypothesis in the context of our country. In November 2011, the Portuguese Government ordered an increase in the NHS co-payments *(“taxas moderadoras”)* for several health services, namely the Public Emergency Departments (ED). This was one of the policies put forward by the Portuguese Government following the Portuguese financial crisis, and had the explicit aim of controlling NHS demand, namely in Hospital EDs where the number of non-urgent visits was close to 40 % of total ED demand [15].

Considering that ADSE beneficiaries have the advantage of resorting to a low-price private alternative within their plan’s network, while single insured NHS patient have to pay the full price of care in the private sector, a policy that increases co-payments for NHS services may induce a larger reduction on demand for Public health services among ADSE beneficiaries compared to single insured NHS patients. Using a detailed database from three different-level EDs, we rely on *a difference-in-differences* approach to test this hypothesis, under the assumption that, during the period of the co-payments’ increase, the main difference between ADSE (our treatment group) and NHS (our control group) sub-populations was the former’s possibility of using their networks’ health services to “escape” from the increase in prices in the public sector.

### Portuguese context

Portuguese have the right to health protection through a NHS, which should be general, universal and almost free of charge, according to patients’ social and economic status. The non-gratuity of the System translates into the existence of co-payments (“taxas moderadoras”) that some patients – those who are not exempt due to their social or economic situation – have to pay when accessing the NHS services.

The Portuguese Emergency Network comprises three levels of care – polyvalent EDs, medical–surgical EDs and basic EDs– which differ in terms of the complexity of the cases they receive, the resources they have (e.g. human resources, lab and imaging exams), the price the Government pays the hospitals for each ED visit and the level of co-payment required to each patient. The polyvalent emergency services are those that receive the most complex patients. Basic emergency services receive only patients with simple cases. Medical–surgical emergency services are at an intermediate level, receiving cases with some complexity, but referring the most complex ones to polyvalent emergency services, according to specific clinical criteria (e.g. a patient presenting in a Basic ED with appendicitis is referred to a medical–surgical ED; a polytraumatized, if taken to a medical–surgical ED, is referred to a polyvalent ED) and pre-determined referral networks (a basic ED always refers to the same medical–surgical ED, which always refers to the same polyvalent ED). The differences in the complexity of the cases treated in each type of ED also translate into differences in the value of the co-payments. On January 1, 2012, co-payments for several health services provided by the Portuguese NHS were significantly increased. For ED, co-payments were increased between 75 and 108 %, depending on the classification (degree of complexity) of the ED: co-payments for basic ED visits were established at €15 (an increase of 75 %), co-payments for medical–surgical ED visits were established at €17.5 (an increase of 103 %) and co-payments for polyvalent ED visits were established at €20 (an increase of 108 %).

One other feature of the Portuguese Health System which is important for our work is the existence of health subsystems, alongside the NHS. These are health insurance schemes that were established before the NHS and that now represent a second layer of health protection for some citizens. Usually these give easy access to a wide range of private providers at a lower price. Naturally, regarding the NHS, these citizens are subject to the same conditions as any NHS patient: they have to pay the services’ co-payments (as long as they are not eligible for exemption) and they have waiting times for outpatient consultation or surgery.

The focus of our work is on the Civil Servants’ health subsystem (ADSE), the largest subsystem in Portugal, covering a population of 1,3 million citizens (more than 10 % of the population). ADSE is funded both by employer (State) and employees’ contributions, with additional transfers from the State budget. Increases in the level of both employer and civil servants’ contributions have recently been made: from an employees’ contribution of 1,5 % of income in the beginning of 2013, the rate has increased to 3,5 % in 2014; and public services started a contribution of 2,5 % of their civil servants’ income in 2011. Due to this increase in public services’ contributions, Government Budget transfers to the ADSE ceased in 2012 and the system became self-sustainable for the first time.^1^ ADSE beneficiaries may access private services by two ways: first, there is a list of private providers with whom ADSE has agreements and where access is almost free (they only have to pay small co-payments); second, they may access other providers that are not in the list, pay the service fee and submit it for reimbursement.

## Methods

Our study used administrative electronic data obtained from three Portuguese hospital EDs in the North of Portugal: a Polyvalent, a Medical-Surgical and a Basic ED, representing therefore all the levels within the Portuguese ED network.

The Hospital de São João is the largest academic hospital in the North of Portugal. Its polyvalent ED has an average volume of 150 000 annual visits and is at the top of the referral network for 41 of the 86 municipalities in the North of the country, covering a population of about 1.7 million. The other two are smaller proximity hospitals, which together serve a population of about 250 000. Hospital Conde de São Bento in Santo Tirso has a basic ED and Hospital São João de Deus in Famalicão has a medical–surgical ED. The three EDs included in our sample account for almost 10 % of the ED visits in Portugal, especially because of HSJ which is one of the hospitals with the highest ED volume in the country.

We compared ED demand made, respectively, by ADSE beneficiaries and NHS patients in the three EDs for a period of 6 months before the co-payments’ change (January to June, 2011) and an equal period after the changes (January to June, 2012). Moreover, since our aim was to measure the effect of the co-payment’s increase, we selected only patients that ought to pay the co-payment (patients who were not exempt from these charges), regardless of their payer (ADSE or NHS). We also excluded from this analysis patients who visited the ED using other health insurance (e.g. private health insured individuals or beneficiaries of private health subsystems such as the Banking Health Subsystem).

For estimating this effect, we resort on a *difference-in-differences* approach.

Consider the following general model, without covariates for the moment:

1 $$ {Y}_i = {\beta}_0 + {\beta}_1{T}_i + {\beta}_2SU{B}_i+\kern0.5em {\beta}_3{T}_i\ast SU{B}_i, $$

where Y is our measure of the ED demand (see below); T is a dummy variable which equals 1 if the ED visits were made in 2012 and 0 if they were in 2011 and captures any aggregate factors that caused changes in ED demand over time; SUB is a dummy variable which equals 1 if ED visits were made by double insured ADSE beneficiaries and 0 for the ED demand by NHS user and captures the differences in ED demand between these populations before the payment increase. The DD estimator,*β*_*3*_, gives the differences in ED demand between ADSE and NHS patients after the policy that increased co-payments.

The DD methodology has been widely used in health economics to estimate the impact of policy interventions, and specifically in studies that had an aim similar to ours: Ikenwilo used a DD framework to assess the effect on NHS utilization of a free dental check-up program in Scotland [16]; Layte et al. and Nolan evaluated a policy of exempting patients over 70 y.o. of GP user charges in Ireland [17, 18]; and Chen and Jin examined whether increased insurance coverage in China led to lower child and maternal mortality and better school enrollment [19], among many others. The main assumption behind the DD methodology is that other changes that have occurred during the period analyzed affect the intervention and control groups equally. Similarly, in our study, any change of utilization of Public EDs by NHS patients and ADSE beneficiaries that is not related to “the double coverage effect” (the existence of a low-price private alternative for civil servants) should be captured by the demand of NHS patients, who don’t have a network of private providers. Therefore, any difference in variation on the ED demand between ADSE beneficiaries and NHS patients, during the period of the increase in co-payments, should reflect the effect of the existence of a (low price) private alternative within the ADSE’s network which could lead to a shift to/from the private sector among these patients, caused by the public co-payments’ increase. Differences in utilization of Healthcare services between NHS and ADSE patients are well documented [9, 10] and even a fairly different average income level is argued to exist between the two groups [20]. Such factors should not bias our results since we studied the demand between 2011 and 2012 and those different utilization patterns already existed in 2011 and are then captured by *β*_2_.^2^

We analyze ED demand using the weekly number of ED visits (our outcome measure), made by each of our groups (NHS and ADSE patients), in each year. We use a negative binomial regression to estimate our model, since our outcomes were counts and their distribution was more overdispersed than would be found in a Poisson distribution [21]. Therefore, the coefficients of interest in eq.1) can be interpreted as the log of the expected number of ED visits per week, with negative values indicating a lower ED demand.

Covariates included demographic variables, such as sex and average age at admission, ED demand related variables, such as the referrals by the Primary Health Care network and the proportion of patients discharged home, and a week variable, to account for seasonal effects (a description of these variables is presented in Appendix 1).

We further extended our main model to account for two factors that may be relevant to our analysis: the patients’ severity when seeking for care and the patients’ distance to ED (as a measure of the distance-cost patients had to incur, on top of the co-payment).

There is extensive evidence that patients’ sensitivity to co-payments differs according to the nature of their health problems [22, 23]; Duarte [23], for instance, found that acute conditions, such as appendicitis or cholecystitis - the typical situations that drive patients into the ED with high-severity conditions – are virtually price-inelastic (ie. their demand does not decrease after co-payment increases). Considering this, we classified ED visits using the Manchester Triage’s color on the admission - “green” and “blue” visits were considered low-severity visits, “yellow” were considered intermediate-severity visits and “orange” and “red” were considered high-severity visits. We excluded the Manchester Triage’s “white” group visits, since they are more difficult to classify according to their price-elasticity, ie., they may vary between a “more price-sensitive” and “less price-sensitive” visits (these are patients who had a formal indication from their discharge doctor to return to the ED in the following days for a reevaluation).

Similarly, there is a vast literature positing a substantial effect of distance-costs on ED demand [24]. In Portugal, where direct costs are small and there is a wide exemption scheme in place, distance costs play an important role in modulating ED demand [25, 26]. For taking these costs into account, we classified ED visits into low, intermediate and high-distance ED demand using tertiles of distance to the ED (measured as the shortest route in Kilometres between the patients’ municipality center and the ED).

STATA Ver.12© was used to estimate the multivariate model. ArcGIS (v.10.0, Environmental Systems Research Institute, CA) was used for distance computation.

## Results

### The data

Table 1 shows the number of ED visits, stratified by insurer (NHS and ADSE) and visit’s severity, for each of the periods we analyzed, and the variations that occurred between 2011 and 2012. From 2011 to 2012, overall ED visits decreased by 9 %, with considerably higher decreases in ADSE beneficiaries’ visits (22 %). Furthermore, this decrease on ED demand was higher for low-severity visits (-25 %) than for intermediate (-21 %) and high-severity visits (-18 %).

**Table 1:** ED visits according to Subsystem (NHS vs. ADSE) and Manchester Triage

	2011		2012	
Number of visits (NHS + ADSE)	48 278		43 940	
YEAR-on-YEAR				-8,96 %
	NHS		ADSE	
	2011	2012	2011	2012
Number of visits	41 292	37 796	3160	2472
YEAR-on-YEAR		-8,47 %		-21,77 %
Low-severity visits	14 981	13 807	1031	775
YEAR-on-YEAR		-7,84 %		-24,83 %
Intermediate-severity visits	21 860	20 240	1753	1390
YEAR-on-YEAR		-7,41 %		-20,71 %
High-severity visits	4451	3749	376	307
YEAR-on-YEAR		-15,77 %		-18,35 %

### The DD model

A summary of our *difference-in-difference* model estimates is presented in Table 2. We test for overdispersion using a LR test on the parameter alpha showing that our ED demand variable is over-dispersed and is not sufficiently described by the simpler Poisson distribution. The full model, presented in Appendix 1, highlights the non-significance of demographic and ED-related covariates that could cause ED demand to change during the period of our analysis. The second row of Table 2 presents the DD coefficient ${\beta}_3$ (an estimate of the effect of having double health coverage, as explained in our methodology), following the policy that increased co-payments for ED. In non-linear models, the interaction terms are not directly interpretable in terms of intensity and statistical significance [27]. For a rigorous analysis, we compute the marginal effects and transform them into semi-elasticities for easiness of interpretation.

**Table 2:** Negative binomial regression – main coefficients

	Coefficient (SE)	IRR (95 % CI)
2012	-0.167 (0.030)***	0.924 (0.868 – 0.983)**
*ADSE*	- 2.666 (0.098)***	0.076 (0.062 – 0.092)***
2012 ∗ *ADSE*	-0.176 (0.038)***	0.839 (0.779 – 0.903)***
alpha	0.004 (0.001)***	
Pseudo R^2^	0.3504	

Note: These are the results of the *negative binomial* regression. The dispersion parameter, *alpha*, is significantly greater than zero based on a likelihood-ratio chi-square test. The number of observations is 104 (number of ED visits/week). Column 2 presents the average marginal effects transformed into semi-elasticities: these give an approximation of how much ED demand is expected to increase or decrease for a unit change in the independent variable. Robust standard errors are shown in parentheses. Column 3 presents the multiplicative effects in IRR. Statistical significance is denoted as follows: **p < 5 %.***p < 1 %

Table 2 shows that the DD coefficient *β*_3_ was statistically significant and negative. The policy of increasing co-payments was associated with a decrease in demand of 18 % among ADSE patients, after controlling for the demand made by NHS patients.

On Table 3, we test whether this differential ED demand among ADSE and NHS patients after the co-payments’ increase was sensitive to the severity of ED visits and the distance patients had to travel to the ED. Our results show that only on low and intermediary-severity demand was the difference on ED demand statistically different between ADSE and NHS patients, after the increase in co-payments; specifically, ADSE patients had a 20 and 15 % reduction in demand in 2012 for low and intermediate-severity conditions, respectively. High-severity conditions reduced 3 %, yet this effect was not statistically significant. Similar results emerge for distance: the decrease in ED demand among ADSE patients was only statistically significant for patients who lived closer to the ED. For these patients, after the increase in co-payments, ED demand reduced 20 %.

**Table 3:** Negative binomial regression according to severity and distance – DD estimators

	Low-severity	Intermediate-severity	High-severity
2012 ∗ *ADSE*	-0.205 (0.058)***	-0.155 (0.045)**	-0.031 (0.087)
Pseudo R^2^	0.3525	0.3555	0.3645
	Low-distance	Intermediate-distance	High-distance
2012 ∗ *ADSE*	-0.192 (0.047)***	-0.201 (0.058)***	-0.094 (0.061)
Pseudo R^2^	0.3708	0.3674	0.3501

Note: these are the results of the *DD* coefficient for ED demand according to ED severity and distance. Rows 2 and 4 present the marginal effects. Robust standard errors are shown in parentheses. Statistical significance is denoted as follows: **p < 5 %.***p < 1 %

## Discussion

In this study, we estimate the effect that the recent increase in co-payments had on the demand for ED care by double insured ADSE (civil servants) patients, who have a private alternative in their plan’s network. To our knowledge, even though co-payments are becoming increasingly widespread among European Health Systems, we are the first to assess the impact of an increase in NHS co-payments on these subpopulations’ demand for health services.

We used a difference-in-differences design to estimate this “double coverage effect”, using the NHS patients as the control group. Our main assumption is that, during the period of the co-payment’s increase, the only difference between NHS and ADSE patients is the latter’s opportunity to use a low-price private provider within their plan’s network, as an alternative to the increase in price in the public sector.

We found that following the increase in co-payments, ADSE beneficiaries had a sizeable decrease on Public ED demand, compared to NHS patients. Furthermore, this decrease was only due to low and intermediary-severity ED visits (“blue”, “green” and “yellow” ED visits, according to the Manchester Triage), which are usually conditions that have a closer substitute in private hospitals and clinics. This strengthens our hypothesis that ADSE beneficiaries shifted their ED demand to private alternatives within their plan’s network following the co-payment increase in public hospitals in 2012. Moreover, we did not find this decrease in demand among emergent ED episodes. Two arguments may explain this finding: firstly, most emergent ED conditions (regardless of the insurance) are transported to the hospital by the National Emergency Medical Service (INEM) that only refers patients to public hospitals. Furthermore, there is extensive evidence [23, 25] that high-severity conditions are virtually price-inelastic, so their demand should not be affected by minor variations in co-payments.

Additionally, we also found that ADSE patients who lived closer to the ED were the ones who had the highest decreases in demand. This may be due to the share that co-payments have in the total cost of the ED visit for these patients (ie. they virtually have no distance-costs) or to the fact that patients who live in the metropolis have arguably more and closer alternatives in the private sector than patients who live in rural areas.^3^

On top of our findings of a decrease on public ED demand, ADSE reported an increase of 12 % in consultations per capita in private providers in 2012 (and more than 20 % in private emergency departments), which reinforces our hypothesis of reallocation of demand from the public to the private sector after the increase in NHS co-payments [28].

Our evidence on opting-out behaviour by double insured ADSE patients is consistent with the work of previous authors and provides another practical example of the Besley and Coate (1991) argument [11]. Our finding of a 20 % decrease in double-insured ADSE demand is sizeable, but is within the range of the estimates of other authors: Sogaard and colleagues [14] report that in Denmark employer-paid health insurances led to a 10 % reduction of public hospitals while Fabbri and Monfardini [12] estimate that double insured Italian patients may reduce public demand by as much as 70 %.

Our findings have some economic and political implications.

**Fig. 1:**
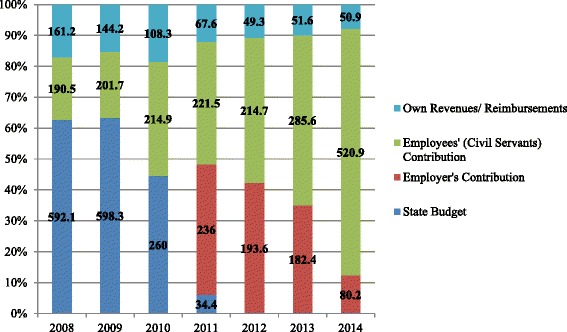
ADSE Funding Structure (data in million euros); source: own elaboration based on ADSE annual reports. Notes: Civil servants’ contributions did not change between 2011 and 2012. They started to increase after 2012: a first increase from 1,5 % to 2,25 % (as a function of income) in August 2013, a second increase in January 2014 from 2,25 % to 2,5 % and from 2,5 % to 3,5 % in May 2014

Firstly, note that, interestingly, this opting out behavior we found following the increase in co-payments for public EDs highlights an overall discussion that is taking place in Portugal over the equity of the entire Health System: if and whenever taxpayer budget helps financing these subsystems, as regularly happened in Greece [1] and Spain [2] and in Portugal until 2012 (Fig. 1), the general population feels this is iniquitous since they are financing the private alternative network ADSE grants to their beneficiaries; if the employees (civil servants) and employer (State) contributions are sufficient for financing the subsystem, ADSE beneficiaries feel this is iniquitous since they are financing both the subsystem and the NHS but increasingly make use of the former and cannot opt out from the latter. Since much of the issue stands along the capability of the civil servants’ subsystem to become self-sustainable (ie., the end of government budget transfers into these systems’ accounting) and, in this sense, dependent on the recent increases in civil servants’ contributions to their subsystem, our findings provide one additional piece of information for the current discussion over the sustainability and future of the civil servants’ health subsystem.

Additionally, our findings emphasize the importance of the existence of a perceived trustworthy alternative, rather than the mere reliance on demand-side measures, in order to successfully redirect patients towards more cost-effective settings. This is extremely important, in light of the recent research that found limited – if any – impact of the increase in co-payments on the general NHS population [25]. This stresses the need for tailoring supply-side measures at the primary health care (PHC) setting and at the PHC-ED gap, as a way of increasing accessibility and freedom of choice within the Portuguese NHS and enhance the patients’ reliance in the alternatives to the ED.

Finally, the bigger decrease among low-cost low-severity ED visits, compared to resource-intensive high-severity ED visits, points towards skimming in the ED market. While for the Health subsystem ADSE the net effect can be null - or even positive if competition leads to a lower ED visit price -, the impact on public hospitals is surely negative for at least two main reasons: firstly, allocative efficiency measures to cope with changes in demand are difficult to implement in the ED setting (e.g. by law, doctors from other departments have to devote a certain amount of their week time to work in the ED), resulting in high fixed costs; secondly, the payment mechanism to hospitals for ED production is a severity-independent flat-fee, which back-of-the-envelope calculations show that does not even accurately represents the costs of an average (severity) ED visit [29]. To the extent that Public EDs have to deal with an average higher case-mix now, this will certainly incur public hospitals into operating with (even higher) negative economic profits in this line of production. This ED skimming calls for changes in the financing of Public EDs, namely by establishing severity and outcome-adjusted payment. Since 2012, the MoH has made public the intention of including such measures in the annual production contracts made with public hospitals, yet their formal incorporation is still lacking.

Nevertheless, our paper has some limitations.

The main limitation of comparing utilization rates before and after the increase in co-payments results from an increase in the number of co-payment exempt citizens in Portugal that occurred simultaneously to the increase in co-payments. However, this should only have an impact in our analysis if non-exempt sub-populations among ADSE and NHS patients increased by different amounts. Considering that the main change in the exemption criteria was a decrease in the wage cut-off for financial exemption [15] and that civil servants have, on average, a higher income level compared to private employees [20], our results may be underestimated (ie. we should find an higher decrease on ED demand among NHS patients just because there were less patients who had to pay the co-payment among NHS patients in 2012). Therefore, this should not change our main finding of a higher decrease on ED demand among ADSE beneficiaries compared to NHS patients.

Furthermore, we did not consider the fact that some patients that visited the ED using the NHS may be enrolled in a private health plan and, in this sense, may be also double insured. If these patients also experienced a “double coverage effect” following the co-payment increase, our findings may be underestimated.

Finally, our study focused on the demand for public EDs in the North of Portugal. While the effect of the financial crisis and the reduction of disposable income were probably higher in this region, the number of private providers is lower compared to Lisbon, for instance. The overall net result and the extent to which they are generalizable to the rest of the country are therefore unknown.

## Conclusions

Following an increase in co-payments for Portuguese Public EDs, we found that civil servants experienced a sizeable reduction on ED demand, compared to single insured NHS patients. During this period, the main difference between these two sub-populations was the former’s possibility of resorting to a low price private alternative within their own supplementary insurance scheme’s network.

In several taxation-based National Health Services, the co-existence of supplementary health subsystems and mutual schemes raises concerns about the overall equity of the Health System, since some citizens subsidize both their Health subsystem and the universal NHS, but increasingly opt out from using the latter. We show that an increase in the direct costs of health within the National Health Service may exacerbate these equity and efficiency implications and should be taken into account in the current discussion over the future of the civil servants’ subsystem.

## Appendix 1

**Table 4:** Variables description

Variable	
2012	=1 if ED visits were made in 2012, 0 otherwise
ADSE	=1 if ED visits were made by double insured ADSE beneficiaries, 0 otherwise
2012 * ADSE	=1 if ED visits were made by double insured ADSE beneficiaries in 2012 (the DiD)
Female	the proportion of ED visits that were made by women (per week)
PHC origin	the proportion of ED visits that were originated from the PHC network (per week)
Discharge Referral	the proportion of ED visits that were discharged home (per week)
Distance	the average distance patients had to travel to the ED (weekly ED demand)
Age	the average ED patients’ age (weekly ED demand)
Week	the number of the week, in each year

**Table 5:** Full model estimates

Variable	B	IRR (95 % CI)
2012	-0.080 (0.032)**	0.924 (0.868 – 0.983)**
ADSE	-2.578 (0.100)***	0.076 (0.062 – 0.092)***
2012 * ADSE	-0.176 (0.038)***	0.839 (0.779 – 0.903)***
Female	-0.016 (0.338)	0.984 (0.508 – 1.913)
Distance	0.004 (0.005)	1.004 (0.995 – 1.013)
Age	0.005 (0.008)	1.005 (0.991 – 1.020)
PHC origin	-1.523 (1.661)	0.218 (0.008 – 5.652)
Discharge Referral	0.536 (0.274)	1.708 (0.998 – 2.924)
Week	-0.002 (0.001)	0.998 (0.779 – 1.001)
Pseudo R^2^	0.3503	
alpha	0.004***	

Note: these are the results of the full model. The second column presents the coefficients directly extracted from the model (not the marginal effects). The last row shows a likelihood ratio test that alpha equals zero--the likelihood ratio test comparing this model to a Poisson model. Statistical significance is denoted as follows: ***p* < 5 %.****p* < 1 %

## Footnotes

1 Please note that this increase in employers’ contribution was a change in the State’s funding mechanism of ADSE with virtually no effect for ADSE users and that the general public was mostly unaware: before 2011, the Government financed the health subsystem, ADSE, directly through State Budget transfers; after 2011, the Government continued financing the health subsystem, but using public services’ (employers’) contributions and direct transfers from the State Budget ceased.

2 To our knowledge, there are no studies that have compared the quality of private and public EDs. There is the perception that private EDs have lower waiting times, at least in part because they have lower demand. However, there are no reasons to believe that any aspect of the quality of care in public or private EDs changed significantly during the period of our analysis.

3 For instance, in Porto, the ten largest private hospitals are, on average, 6,5Km distant from the public ED. The average distance that patients in our “low-distance” and “intermediate-distance” groups had to travel was 4Km and 9Km, respectively, which makes the private providers an attractive distance-cost alternative to the public hospital for ADSE patients.
